# Innate sensitivity to stress facilitates inflammation, alters metabolism and shortens lifespan in a mouse model of social hierarchy

**DOI:** 10.18632/aging.102440

**Published:** 2019-11-09

**Authors:** Maryia Bairachnaya, Oryan Agranyoni, Marina Antoch, Izhak Michaelevski, Albert Pinhasov

**Affiliations:** 1 Department of Molecular Biology, Ariel University, Ariel 40700, Israel; 2 Department of Pharmacology and Therapeutics, Roswell Park Cancer Institute, Buffalo, NY 14263, USA

**Keywords:** social behavior, stress sensitivity, aging, chronic inflammation, IGF-1

## Abstract

It is known that stress alters homeostasis and may lead to accelerated aging. However, little is known about the contribution of innate susceptibility to stress to the deterioration of physiological functions, acceleration of aging and developing of age-related diseases. By using socially-submissive stress susceptible (Sub) and socially-dominant stress resilient (Dom) selectively bred mouse model we observed a marked reduction in the lifespan of both male and female Sub mice. We found that innate susceptibility to stress correlates with chronic inflammation, development of splenomegaly and a significant increase in the levels of circulating pro-inflammatory cytokines IL-1β and IL-6. Furthermore, Sub mice showed a marked hypoglycemia, reduction of insulin levels, increase in GSK3 activity and elevation of IGF-1 serum levels, as well as low skin surface temperature and body weight. Interestingly, lifelong exposure of Sub mice to chronic mild stress did not further reduce their lifespan, indicating a high level of intrinsic stress. Taken together, our data reveal that social submissiveness coupled with innate stress sensitivity coincides with inflammation, leading to the deterioration of physiological functions and early aging independent of whether an individual is exposed to stress or not.

## INTRODUCTION

Biological aging is defined by a progressive and generalized impairment of physiological functions, which results in a decreased adaptive capacity, a reduced resistance to harmful environmental factors and an increased threat of disease acquisition. Frequently, external factors contribute to the deterioration of physiological functions and even accelerate the rate of aging. Social stress is increasingly being attributed to such factors affecting an individual’s health and quality of life, contributing to aging and aging-related diseases [1, 2].

Animal studies have shown that social defeat affects physiological parameters, leading to alterations in social behavior, anhedonia, changes in drug preference and disease susceptibility [3–5]. It has also been demonstrated that social stress has a much stronger impact on animal physiology and behavior than normally used stressors such as restraint, electric shock, and chronic mild stress [6]. In addition, it has also been shown that life-long social defeat significantly affects survival [7].

However, the response to social stress varies among populations. This variation is mainly due to significant genetic heterogeneity [8, 9]. Innate resilience or sensitivity to stress may have a substantial influence on normal physiological processes, including immune response and metabolism, and as a consequence on aging and related diseases.

We hypothesize that sensitivity to stress, characterized by the hyperactivity of the hypothalamus pituitary adrenal (HPA) axis, determines the susceptibility to inflammation-driven diseases and underlies the tendency of increased catabolic processes, which lead to the organism’s vulnerability and accelerated rate of senescence.

In this study we demonstrate, using unique mice models of social dominance and social submissiveness, that the inherent subordinate rank associated with an innate stress sensitivity is characterized by a decreased life expectancy, an implicated development of permanent inflammation, a marked splenomegaly, hypoglycemia and a decreased body weight. These findings reveal the impact of social stress and individual adaptation capability on longevity and aging-related diseases.

## RESULTS

### Stress vulnerable Sub mice show a significantly shorter lifespan in comparison to their stress resilient dominant counterparts

Selectively bred Dom and Sub as well as outbred Sabra (background strain, BS) mice were housed in the absence of obvious external stressful interventions for their entire life. The survival time of all the animal groups was monitored and compared with each other.

**Figure 1:**
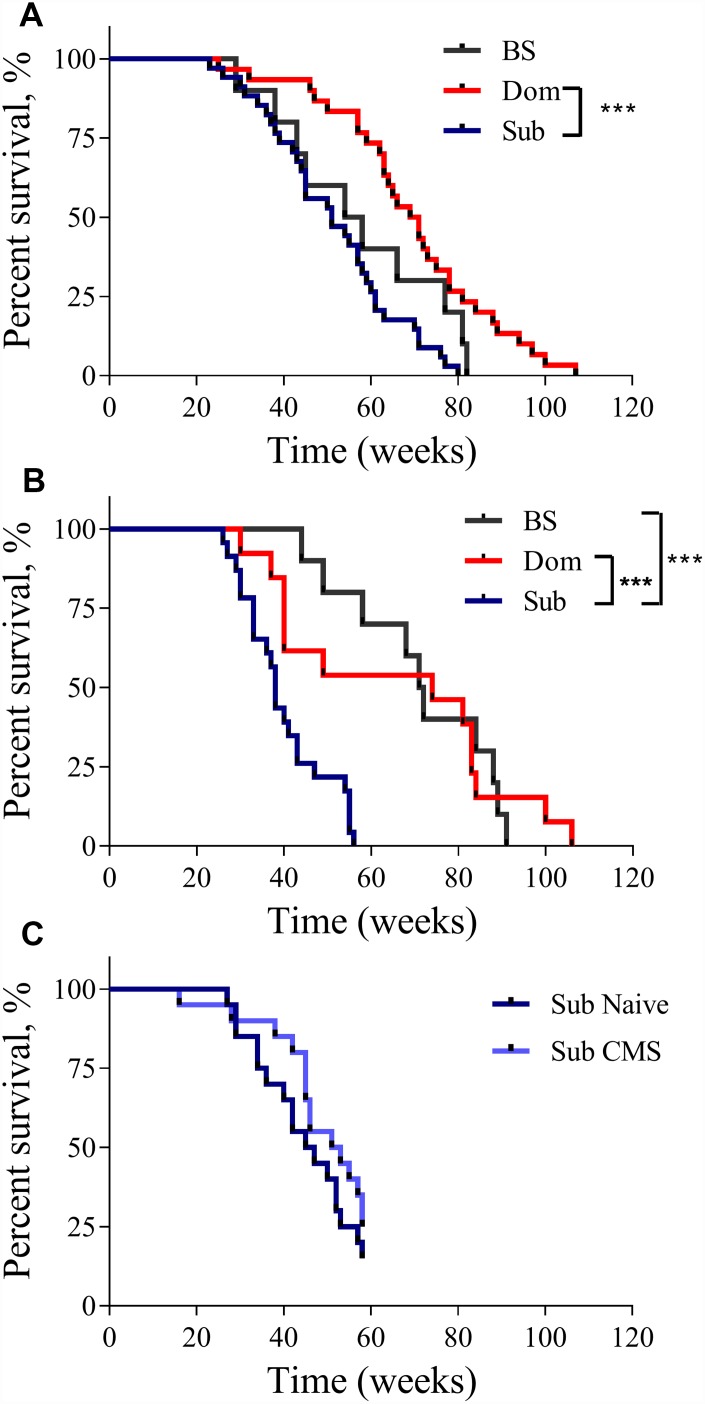
**The lifespan of Dom, Sub and BS mice.** The Kaplan-Meier survival curves of Dom, Sub and BS mice in (**A**) males (log-rank test, *p*<0.001;n=30 for Dom and n=34 for Sub, and n=10 for BS), and (**B**) females (log-rank test, *p*<0.05; n=13 for Dom and n=23 for Sub, and n=10 for BS), (**C**) Sub naïve (n=20) and exposed to CMS (n=20) males. * - *p*<0.05, *** - *p*<0.001.

The analysis of the Kaplan-Meier survival curves showed that the lifespan of the Dom animals was significantly longer than that of the Sub animals. This was true for both males (Figure 1A, log-rank test, χ^2^ = 18.14 df=1, *p*<0.001; n=30 for Dom, n=34 for Sub) and females (Figure 1B, log-rank test, χ^2^ =10.92 df=1, *p*<0.01; n=13 for Dom and n=23 for Sub). The visual difference in lifespan between Dom and BS males was not found to be statistically significant (Figure 1A, log-rank test, χ^2^ =2.951 df=1, p=0.0858; n=30 for Dom, n=10 for BS). No significant differences were detected between Sub and BS males (Figure 1A, log-rank test, χ^2^ =2.625 df=1, *p*=0.1052, n=34 for Sub, and n=10 for BS). In contrast, Sub female mice had a significantly shorter lifespan in comparison to BS females (Figure 1B, log-rank test, χ^2^ =19.62 df=1, *p*<0.001; n=23 for Sub, and n=10 for BS). In addition to this remarkable decrease in the maximum lifespan, the survival probability at the median lifespan of Sub mice compared to Dom declined by 27.1% and 48.6% for males and females, respectively. Furthermore, the Sub phenotype had a significantly higher hazard ratio than the Dom phenotype, this being 2.583 in males (Hazard ratio (log-rank), 95% CI = 1.517 to 4.398) and 2.581 in females (Hazard ratio (log-rank), 95% CI = 1.327 to 5.019). Intriguingly, the exposure to chronic mild stress (CMS) did not affect the survival probability of Sub male mice (Figure 1C), despite us having previously shown that these mice are sensitive to CMS [10].

### Stress vulnerable animals show marked alterations in physiological and metabolic parameters of reduced body weight, blood glucose level and skin surface temperature

Aging-related changes represent a cellular metabolic deterioration leading to the dysregulation of essential physiological functions. It has been previously reported that many physiological parameters change with age. These include body weight, blood glucose [11] and body temperature [12]. Therefore, we have implemented a series of tests that were performed in Dom and Sub mice over the period of their entire lifespan. The assessment time was limited by the maximum lifespan of the Sub females (56 weeks).

**Figure 2:**
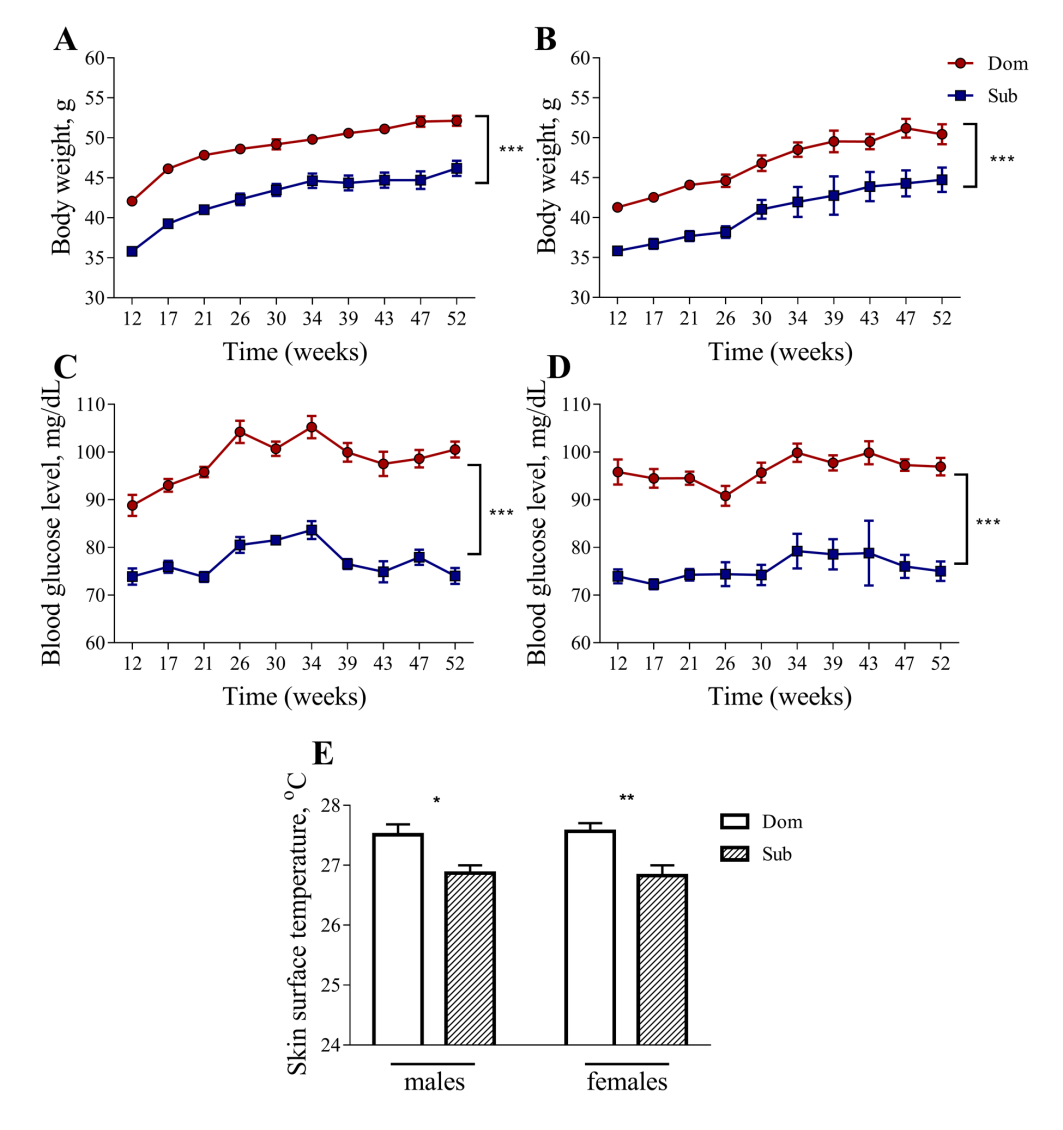
**Body weight, blood glucose level and skin surface temperature in Dom and Sub mice.** The body weight (males (**A**), females (**B**)) and blood glucose level (males (**C**), females (**D**) are significantly higher in Dom mice (two-way ANOVA, *p*<0.001). A significant decrease in body surface temperature (**E**) was observed in stress sensitive Sub male (Student unpaired two-tailed *t*-test, t=2.653, *p*<0.05) and female (Student unpaired two-tailed *t*-test, t=3.335, *p*<0.01) mice in comparison to their Dom counterparts. Dom males n=30, females n=15; Sub males n=20, females n=16. * - *p*<0.05, ** - *p*<0.01. Error bars indicate SEM.

Despite having a similar food consumption to Dom mice (data not shown), both male and female Sub mice had a significantly lower body weight in comparison to their Dom counterparts (Figure 2A and 2B). The Sub mice also showed a significant decrease in blood glucose levels, indicating marked hypoglycemia (Figure 2C and 2D). Furthermore, the Sub animals exhibited a marked hypoglycemia from an early age, showing a significant decrease in blood glucose levels from the age of 12 weeks and throughout the study, in both males (Figure 2C, two-way ANOVA, *p*<0.001, n=30 for Dom, n=20 for Sub) and females (Figure 2D, two-way ANOVA, *p*<0.001, n=15 for Dom; n=16 for Sub). The Sub mice were also characterized by a decreased body weight in both males (Figure 2A, two-way ANOVA, *p*<0.001, n=30 for Dom, n=20 for Sub) and females (Figure 2B, two-way ANOVA, *p*<0.001, n=15 for Dom; n=16 for Sub).

The body temperature of Sub mice was also significantly lower than that of Dom mice, suggesting a decrease in their metabolic activity (Figure 2E, 27.51±0.18 °C for Dom males, 26.87±0.13 °C for Sub males, 27.57±0.14 °C for Dom females, 26.83±0.17 °C for Sub females).

### Insulin and IGF-1 signaling in Sub mice is diminished

**Figure 3:**
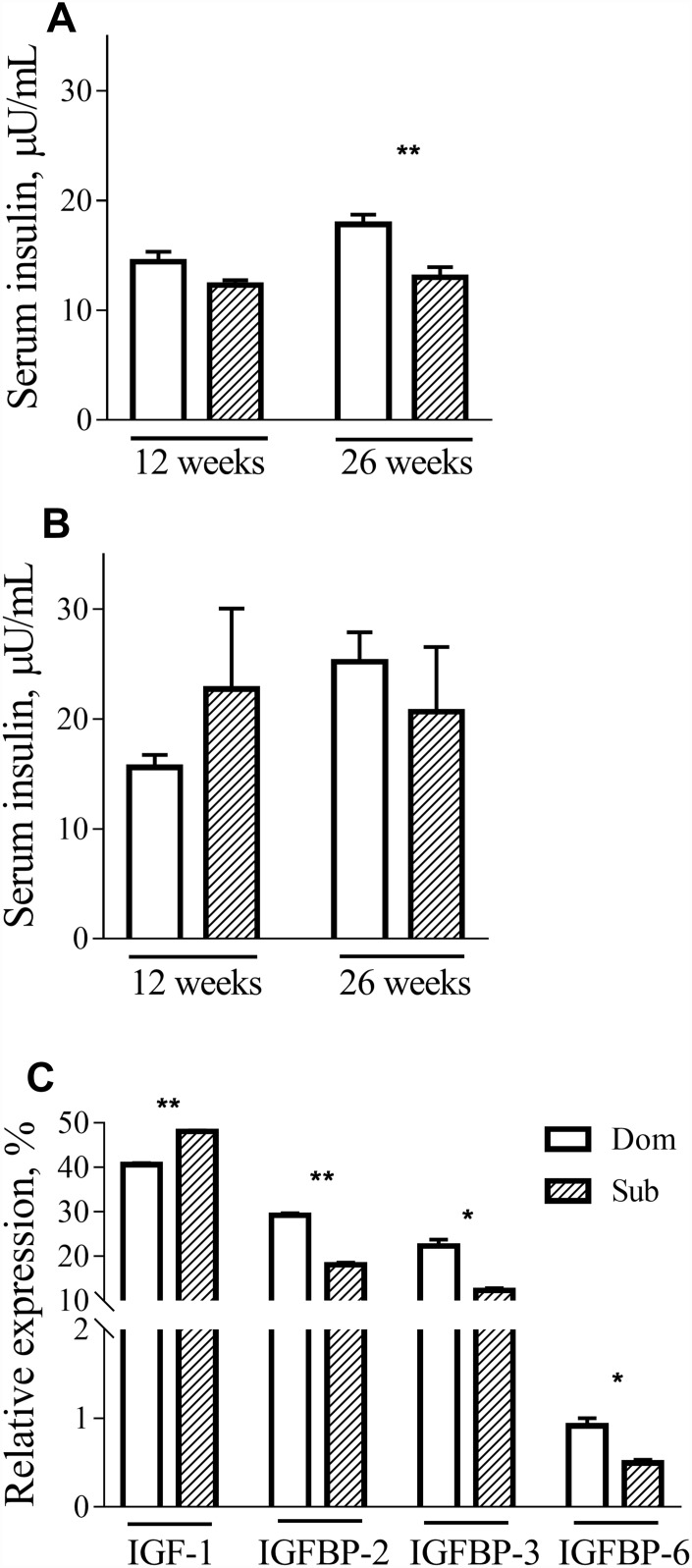
**Insulin, IGF-1 and IGFBPs circulation levels.** The insulin levels in male (**A**) and female (**B**) Dom and Sub mice at the age of 12 weeks (Dom males n=5, females n=5; Sub males n=5, females n=6) and 26 weeks (Dom males n=5, females n=4; Sub males n=5, females n=5). In 26-week-old Sub males the fasting insulin level was significantly lower than in Dom (t=3.803, *p*<0.01). (**C**) IGF-1, IGFBP-2, IGFBP-3 and IGFBP-6 serum levels in Dom and Sub male mice. In the serum of Sub mice a significant elevation of IGF-1 (t=20.11, *p*<0.01) and a significant reduction of its binding proteins IGFBP-2 (t=17.21, *p*<0.01), IGFBP-3 (t=6.474, *p*<0.05) and IGFBP-6 (t=4.569, *p*<0.05) was detected (n=5 per group). * - *p*<0.05, ** - *p*<0.01. Student unpaired two-tailed *t*-test was used. Error bars indicate SEM.

To further investigate the metabolic alterations in Sub mice, we measured the serum levels of insulin in Dom and Sub mice at different ages. Although no differences in insulin levels during fasting were detected in male mice at 12 weeks of age, difference became prominent at 26 weeks of age (Figure 3A, *t*-test, t=3.803, *p*<0.01; n=5 for each group). In females, there was no significant difference between serum insulin levels in both age groups (Figure 3B).

These findings prompted us to study the expression of the IGF-1 gene, which is involved in different signal transduction pathways, including those of growth, inflammation and survival. Although no difference in mRNA expression in the liver was observed between Dom and Sub animals (data not shown), a significant elevation of IGF-1 (*t*-test, t=20.11, *p*<0.01) and a significant reduction of its binding proteins IGFBP-2 (*t*-test, t=17.21, *p*<0.01), IGFBP-3 (*t*-test, t=6.474, *p*<0.05) and IGFBP-6 (*t*-test, t=4.569, *p*<0.05) was detected in the serum of Sub mice (Figure 3C).

**Figure 4:**
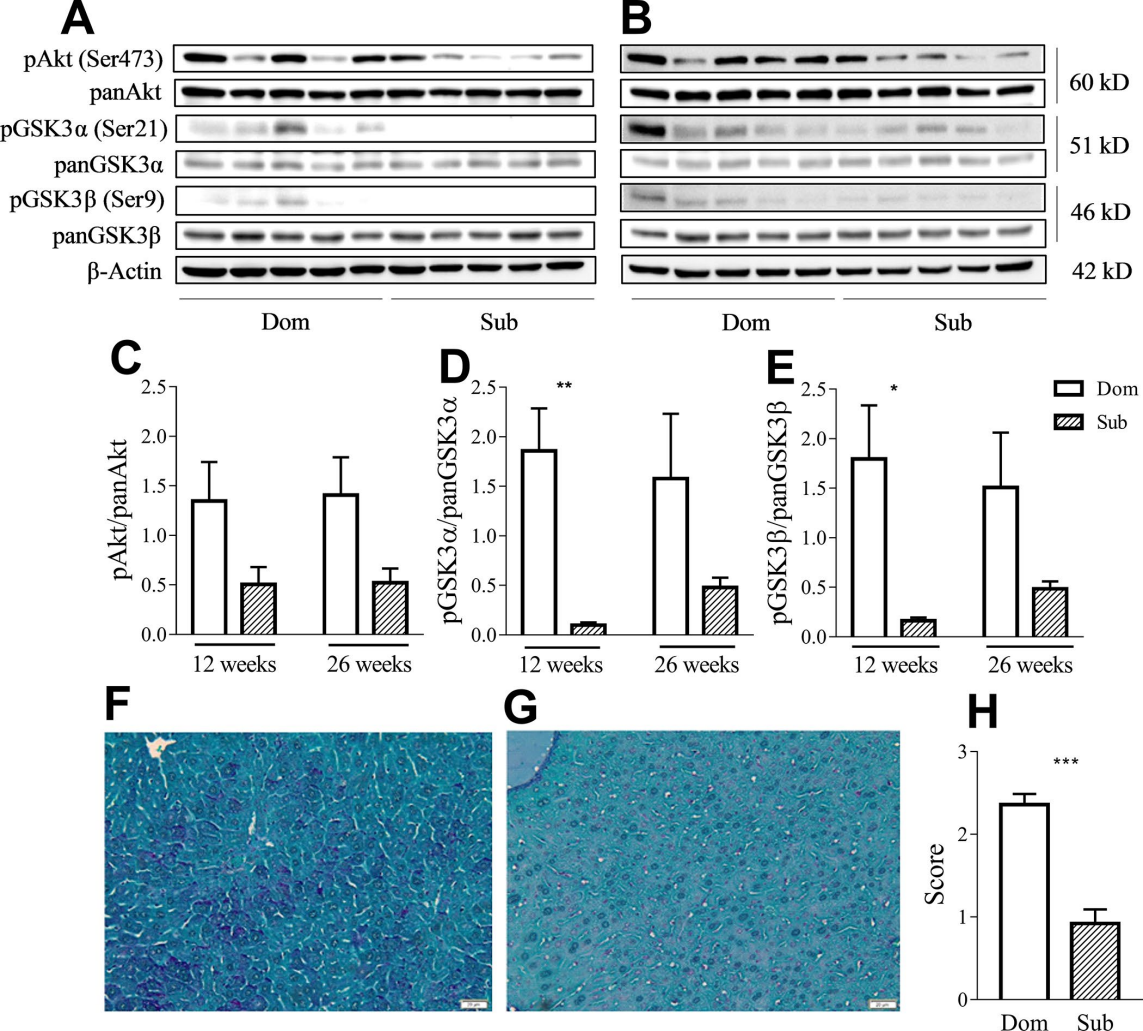
**Sub mice exhibited decreased Akt and GSK3 phosphorylation and glycogen content in liver.** Western-blot analysis of phospho- and pan-Akt and GSK3 levels in the liver of Dom and Sub mice at the age of 12 (**A**) and 26 weeks (**B**). (**C**) Phosphorylation level of Akt (Ser473) in Dom and Sub mice at the age of 12 and 26 weeks (n= 5 for each group). (**D**) The phosphorylation level of GSK3α (Ser21) in Sub mice was significantly decreased at the age of 12 weeks (Student unpaired two-tailed *t*-test, t=4.091, *p*< 0.01; n=5 for each group). (**E**) The phosphorylation level of GSK3β (Ser9) in Sub mice was significantly decreased at the age of 12 weeks (Student unpaired two-tailed *t*-test, t=3.012, *p*<0.05; n= 5 for each group). The amount of glycogen in the liver of Dom (**F**) and Sub (**G**) mice, PAS staining, scale bar - 20μm. (**H**) PAS staining revealed reduced level of glycogen in liver of Sub mice (Mann-Whitney test, *p*<0,001). The staining intensity of PAS staining was scored as 0 (0-25%), 1 (26-50%), 2 (51-75%), or 3 (76-100%) according to the percentage of positively stained cells. * - *p*<0.05, ** - *p*<0.01, *** - *p*<0.001. Error bars indicate SEM.

Having considered that GSK3 is an important factor in the inflammatory response [13] and that its activity is correlated with aging, we evaluated the phosphorylation levels of both its isoforms as well as of its upstream regulator, protein kinase B (Akt), in liver of Dom and Sub mice at the age of 12 (Figure 4A) and 26 weeks (Figure 4B). Despite the presence of a trend of the reduction of the phosphorylation levels of Akt (Ser473) in Sub mice versus their Dom counterparts, the statistical analysis (*t*-test) did not reveal any significant difference (Figure 4C). However, the Sub mice showed a significant decrease in the phosphorylation levels of both isoforms of GSK3 α/β (Ser21/9) at the age of 12 weeks (Figure 4D–4E), further suggesting an enhanced pro-inflammatory profile in these mice. Moreover, Periodic Acid Schiff (PAS) staining of the liver tissue (Figure 4F–4G) revealed significantly lower glycogen levels in the liver of Sub mice in comparison to their Dom counterparts (Figure 4H, Mann-Whitney test, *p*<0.001).

Thus, the observed increase in the effective IGF-1 concentrations may be due to the failure of insulin signaling downstream, as observed by the elevation of GSK3 activity.

### Innate stress susceptibility of Sub mice correlates with chronic inflammation and splenomegaly

**Figure 5:**
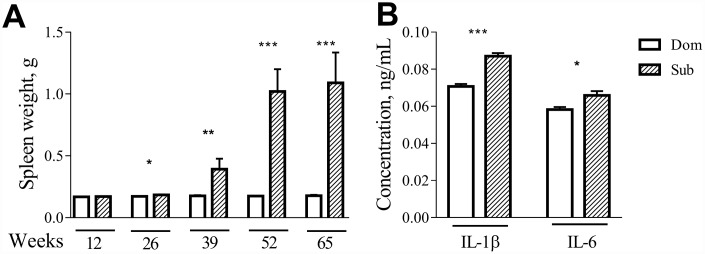
**Age-dependent splenomegaly and increased pro-inflammatory status in Sub mice.** A significant increase in spleen weight was observed in stress sensitive male animals at the age of 26 weeks (t =2.408, *p*<0.05; n=13 for each strain), 39 weeks (t=3.038, *p*<0.01; n=10 for Dom, n=7 for Sub), 52 weeks (t=11.82, *p*<0.001; n=8 for Dom, n=2 for Sub) and 65 weeks (t=7.072, *p*<0.001; n=16 for Dom, n=5 for Sub). The serum levels of pro-inflammatory cytokines (**A**) IL-1β (t=7.611, *p*<0.001) and (**B**) IL-6 (t=2.644, *p*<0.05) in 12-week-old male Dom and Sub mice (n= 21 for Dom, n= 26 for Sub). * -*p*< 0.05, ** - *p*<0.01, *** - *p*<0.001. Student unpaired two-tailed t-test was used. Error bars indicate SEM.

Visual inspection during the postmortem autopsy revealed that the aged Sub mice had an overt splenomegaly. Therefore, we performed a systemic assessment of the spleen weight in male and female mice of different ages. While no differences were observed in the case of 12-week-old mice, at older ages (26, 39, 52 and 65 weeks) the Sub mice developed a significant splenomegaly in comparison to their age-matched Dom counterparts (Figure 5A; 26 weeks (t =2.408, *p*<0.05), 39 weeks (t=3.038, *p*<0.01), 52 weeks (t=11.82, *p*<0.001) and 65 weeks (t=7.072, *p*<0.001)). The same phenomenon was observed in the case of female mice (data not shown).

Since changes in splenic morphology often correlate with alterations in immune functions, we assessed the levels of circulating pro-inflammatory cytokines in serum of Dom and Sub mice at the age of 12 weeks. Even though none of the groups received inflammatory injury, a significant upregulation of interleukins IL-1β was observed in the Sub mice group (Figure 5B, *t*-test, t=7.611, *p*<0.001) and IL-6 (Figure 5B, *t*-test, t=2.644, *p*<0.05) was observed in the Sub mice group.

## DISCUSSION

By using selectively bred socially-submissive stress vulnerable (Sub) and socially-dominant stress resilient (Dom) mice, we have demonstrated that innate susceptibility to stress induces chronic inflammation. Chronic inflammation, in turn, disrupts regulation of anabolism, resulting in the development of age-related pathologies and decrease of life longevity. Moreover, innate susceptibility to stress inevitably accelerates aging, this occurring irrespectively of whether an individual is exposed to stress or not.

We have previously shown that Sub mice are characterized by a high sensitivity to various stressogenic triggers, including acute [14], chronic mild (CMS) [10], social [15, 16] and prenatal [17, 18] stresses. At physiological and biochemical levels, this stress sensitivity is underlined by the hyper-activation of the HPA axis, resulting in elevated levels of stress- induced corticosterone and in the decreased expression of glucocorticoid receptor (GR), leading to an altered negative feedback regulation of the HPA axis activity [18].

Animal studies, as well as human observations, suggest that the altered HPA axis activity may lead to inflammation [1, 19, 20] and accelerated aging [21–23]. Having been inspired by these findings, we measured the longevity of stress-sensitive Sub and stress-resilient Dom male and female mice. We found that both naïve Sub males and females exhibit a significantly shorter lifespan in comparison to their Dom counterparts. Hence, this study clearly demonstrates that innate stress may lead to an acceleration of aging. Phenotypic postmortem observations revealed a marked spleen enlargement in Sub males and females, being in accord with the findings of another study showing a link between splenomegaly and social defeat [24]. Splenomegaly may indicate a possible impairment of the immune system reactivity. In line with this evidence, blood analysis revealed significantly elevated levels of pro-inflammatory cytokines IL-1β and IL-6 in Sub mice in comparison to their Dom counterparts. These findings further support the role of innate stress sensitivity in chronic inflammation.

A growing number of studies provide a functional link between inflammatory processes and the alteration of metabolism [25, 26]. It has been shown that increased concentrations of IL-1β and IL-6 contribute to the weakening of insulin signaling, presumably leading to insulin resistance [27–29]. Notably, a recent study has proposed the existence of a linking bridge between the IL-1β pathway and the related reduction in the activity of Akt [30], a crucial downstream effector of insulin signaling. In this study, we show that the insulin level in Sub mice is markedly lower than in their Dom counterparts. The reduced body weight observed in the Sub mice may serve as additional evidence of the inflammation-mediated anabolism deterioration. A detailed analysis revealed that the reduced level of insulin in Sub mice was accompanied by a strong reduction of Akt phosphorylation, an essential factor of anabolism regulation. Moreover, the reduction of Akt mediated signaling observed in the Sub mice resulted in the enhancement of glycogen synthase kinase-3 (GSK3) activation and reduction of glycogen levels, presumably, due to the impairment of glycogenesis. It should be underlined that insulin shares multiple metabolic and functional effects with IGF-I [31, 32]. Both insulin and IGF-1 activate Akt which phosphorylates serine 9 on GSK3β resulting in its inhibition [33, 34]. Decrease in GSK3β activity in turn leads to the reduced phosphorylation of glycogen synthase, promoting its activation and as a consequence in an increased glycogen synthesis. In addition, activated GSK3β is important for pro-inflammatory cytokine production [35], including IL-1β which is upregulated in Sub mice.

There is an increasing amount of evidence indicating a strong role of the IGF-1 mediated pathway in the control of aging and life longevity. Considerable recent research has focused on various laboratory animal models, demonstrating the correlation between decrease of IGF-1 signaling and an increased lifespan [36–42]. In a strong agreement with these findings, Sub mice, who have exhibited higher levels of IGF-1, have lived less in comparison to their Dom counterparts. Interestingly, despite the elevated IGF-1 serum levels, no differences in liver mRNA expression were observed between Dom and Sub animals (data not shown). Remarkably, we furthermore detected a significant reduction of IGFBP-2, IGFBP-3 and IGFBP-6 in the Sub mice, known to reduce the bio-availability of IGF-1 preventing potential interactions of IGFs with the Insulin Receptor [43]. Thus, the relatively high IGF-1 serum levels together with down-regulation of IGFBP in Sub mice, may be a prerequisite to hypoglycemia. Therefore, we assume that the increased bio-availability of IGF-1 in Sub mice is mediated by the reduction of IGFBP activity, which presumably occurs due to the decrease of insulin signaling. Hence, these results further indicate that the innate stress observed in Sub mice underlies the enhanced inflammation and, as a consequence, the shorter lifespan through Insulin/IGF-1 mediated signaling.

Furthermore, the significant finding of this study is related to the interaction between the response to stressogenic factors and innate stress susceptibility. As clearly shown by our results, the impact of exposure to CMS does not affect longevity in stress-sensitive individuals (Figure 1C). Our findings thus support the notion that predisposition to stress sensitivity is a sufficient condition for aging acceleration and serves as an inevitable factor leading to allostasis and the gradual augmentation of stress-coupled inflammatory processes.

In conclusion, in this study we present several convincing arguments linking innate stress to inflammation, impaired Insulin/IGF-1 signaling, aging-related disorders and accelerated aging. Moreover, we prove that innate susceptibility to stress inevitably accelerates aging whether the individual is exposed to stress or not.

## MATERIALS AND METHODS

### Animals

Dominant and submissive mice strains were originally derived from the Sabra mouse strain based on selective breeding and Dominant-Submissive relationship food competition test as described below.

The animals were housed under a 12-hours of light and 12-hours of dark schedule with standard laboratory chow and water being available ad libitum. All experiments were conducted in compliance with the Animal Care and Use Committee at the Ariel University (protocol number IL-131-04-17).

### Dominant-Submissive Relationship Test (DSR test)

Social interaction phenotypic screening was performed using a DSR test, which allows differentiation between dominant and subordinate animals by the result of competition for food [15, 44]. The animals that spent more time at the feeder were considered dominant. The DSR apparatus, made from Plexiglas, consisted of two identical chambers joined by a tunnel. A 0.5 cm diameter hole was cut at the bottom center of the tunnel. A self-refilling feeder was connected to the tunnel, allowing a constant supply of sweetened milk (3% fat, 10% sugar). The tunnel had narrow slits cut on both sides of the feeder for easy gate insertion and removal. This way, the paired mice had an equal starting position at the beginning of each session. DSR tests were carried out for five consecutive days per week for a two-week period. The animals were food-deprived for 16 hours prior to testing with water being available *ad libitum*. The tests were performed on pairs of weight-matched mice normally housed in different cages. Milk drinking times were manually recorded during each 5-minute DSR session.

### Assessment of physiological parameters

Body weight was measured once a week throughout the study using standard laboratory scales. Animals that lost 20% of their initial weight were euthanized.

Blood glucose measurement was performed using an Accu-Chek Performa apparatus (Roche, Germany) once a month. All measurements were performed between 8-9 AM after overnight fasting.

Skin surface temperature was measured using a non-contact infrared thermometer.

### Chronic mild stress (CMS) exposure protocol

Sub animals were randomly assigned to control and experimental groups. Control mice remained in their original social housing conditions, while experimental animals were exposed daily to a randomly selected stressor in a separate room. CMS procedures were performed for 3 weeks 6 days a week with a 9-10 week interval between the sessions for a 12 month period. The following stressors were used: 1) food deprivation: animals were denied access to food for 8 hours, with access to water being *ad libitum*; 2) night illumination (constant light): animals were placed under illumination equivalent to that of the colony room (200 lux) during the colony’s 12 hour cycle of darkness (19:00-07:00); 3) cage tilt: the cages were placed at a 30° angle during normal rest hours (09:00-17:00); 4) tail pinch: a plastic clothespin was attached to the base of each mouse’s tail for 15 minutes; 5) forced swim: the mice were placed in a 20 cm deep pool of water (25°C) for 5 minutes; 6) cage overcrowding: eight mice were housed in a standard cage during normal rest time (09:00-17:00); 7) wet cage: 250 ml water was added to the animals bedding. After eight hours (09:00-17:00), the bedding was replaced by a dry one; 8) empty cage: the mice were housed in a cage without bedding for eight hours (09:00-17:00).

### Measurement of serum concentrations of insulin using ELISA

Prior to blood collection (via cardiac puncture) animals were food deprived (water *ad libitum*) for 16 hours. The serum concentrations of insulin were measured using a commercially available ELISA kit (Mercodia, Cat No. 10 1247 01, Uppsala, Sweden) according to the manufacturer’s protocol. The serum concentrations of insulin were calculated from a calibration curve generated for each experiment. All reactions were run in duplicate.

### Measurement of IGF-1 and IGFBPs serum concentrations using an antibody-based protein array

An antibody-based protein array (R&D Systems, Cat. No. ARY013, Minneapolis, Minnesota, United States) was used to screen for serum differences in IGF-1 and IGFBP expression between Dom and Sub mice according to the manufacturer’s protocol. An average signal of pixel density from the duplicate spots/adipokine was determined using ImageQuant TL software. The intensity was normalized by control reference values spotted on each membrane. The relative intensity of the reference values (three inside control duplicates in each membrane) was included in the densitometry calculations.

### PAS staining

Dissected livers were fixed in 10% buffered formalin (Sigma-Aldrich, Cat. No. HT501128, St. Louis, Missouri, United States) overnight, embedded in paraffin and then sectioned in to 5 μm sections. Glycogen content was assessed by staining the liver sections with a periodic acid-Schiff (PAS) staining kit (Abcam, Cat. No. ab150680, Cambridge, United Kingdom) according to the manufacturer’s protocol. Briefly, the sections were sequentially incubated with periodic acid for 10 min, washed with water, incubated in the Schiff’s solution for 30 min, washed in water for 1 min, stained with hematoxylin for 3 min, dehydrated with alcohol-xylene and then mounted with Entellan® new mounting (Sigma-Aldrich, Cat. No. 107961, St. Louis, Missouri, United States). Images were then taken using an Olympus BX53 light microscope (Olympus; Tokyo, Japan). The amount of PAS-positive staining (deep magenta color) was assessed by grading the sections from 0 to 3 according to the percentage of positively stained cells, as follow: 0 (0-25%), 1 (26-50%), 2 (51-75%) and 3 (76-100%).

### Western blot analysis

Liver protein lysates were prepared using a RIPA buffer, supplemented with Complete protease inhibitor (dilution 1:25, Roche, Cat. No. 1 838 145, Basel, Switzerland), Phosphatase Inhibitor Cocktail III (Sigma–Aldrich, Cat. No. P0044, St. Louis, Missouri, United States) and 0.1% Triton X-100 (Sigma–Aldrich, Cat. No. T-8787, St. Louis, Missouri, United States). Laemmli sample buffer (Bio-Rad, Cat. No. 161- 0737, Hercules, California, United States) supplemented with β-mercaptoethanol (Sigma-Aldrich, Cat. No. M3148, St. Louis, Missouri, United States) was added to the tissue lysates and boiled for 5 min. 20 μg proteins were loaded per lane and separated by 10% SDS-PAGE gel electrophoresis. The proteins were then transferred onto a nitrocellulose membrane. The membranes were then blocked with 5% BSA in TBST and then incubated with the primary antibodies overnight at 4°C. The following antibodies were used (Cell Signaling Technology, Inc., Danvers, Massachusetts, United States): phospho-Akt (Ser473, Cat. No. 4060), pan Akt (Cat. No. 4691), phospho-GSK-3α/β (Ser21/9, Cat. No. 9331) and GSK-3α/β (Cat. No. 5676). Anti-β-Actin (Sigma- Aldrich, Cat. No. a1978, St. Louis, Missouri, United States) was used as a loading control. The membranes were subsequently washed in TBST and then incubated with secondary antibodies, conjugated with Horseradish Peroxidase, for an hour with shaking at room temperature. Then, the membranes were covered with a chemiluminescent substrate Optiblot ECL Detect Kit (Abcam, Cat. No. ab133406, Cambridge, United Kingdom) and the proteins were detected using the enhanced chemiluminescence. A densitometric analysis was performed with ImageJ software.

### Statistical analysis

The data presented were expressed as mean ± S.E.M. All statistical analyses were performed using the GraphPad Prism software (version 7; San Diego, California, USA). The survival curves were analyzed using the Kaplan-Meier log-rank test. The statistical differences between the groups were tested using an unpaired two-tailed Student *t-*test, a Mann-Whitney test or a two-way analysis of variance (ANOVA), followed by a Bonferroni post-hoc test. A difference of *p*<0.05 in the mean values was considered statistically significant.
